# Supplementary material for: Examining the Factor Structure of the Pittsburgh Sleep Quality Index in a Multi-Ethnic Working Population in Singapore
Source: Int J Environ Res Public Health. 2019 Nov 20;16(23):4590. doi: 10.3390/ijerph16234590 (PMC6926964; doi:10.3390/ijerph16234590)
Supplement: Supplementary file 1 [file ijerph-16-04590-s001.pdf]

Table S1. Correlations between PSQI components and overall scale

	**Correlation of item with overall scale**	**Cronbach's α if the item is eliminated**
Timepoint 1		
Subjective sleep quality	0.640	0.555
Sleep latency	0.638	0.555
Sleep duration	0.575	0.582
Habitual sleep efficiency	0.463	0.626
Sleep disturbances	0.560	0.576
Sleep medication use	0.404	0.645
Daytime dysfunction	0.581	0.580
Cronbach's α	0.627	
Timepoint 2		
Subjective sleep quality	0.634	0.546
Sleep latency	0.658	0.535
Sleep duration	0.515	0.596
Habitual sleep efficiency	0.469	0.614
Sleep disturbances	0.596	0.563
Sleep medication use	0.410	0.635
Daytime dysfunction	0.578	0.571
Cronbach's α	0.619	
Timepoint 3		
Subjective sleep quality	0.682	0.530
Sleep latency	0.669	0.537
Sleep duration	0.575	0.578
Habitual sleep efficiency	0.459	0.623
Sleep disturbances	0.569	0.581
Sleep medication use	0.344	0.662
Daytime dysfunction	0.580	0.576
Cronbach's α	0.624	

Table S2. Sensitivity analysis: Factor Matrix for the two-factor solutions at each timepoint for female participants only.

	**Perceived sleep quality**	**Sleep efficiency**
Timepoint 1		
Subjective sleep quality	.75[a]	.31[f]
Sleep latency	.79[a]	-.05[f]
Sleep duration	.18[f]	.82[a]
Habitual sleep efficiency	-.06[f]	.80[a]
Sleep disturbances	.64[b]	-.13[f]
Sleep medication use	.40[e]	.12[f]
Daytime dysfunction	.69[b]	.18[f]
Percentage of total variance, %	34.7	18.9
Timepoint 2		
Subjective sleep quality	.74[a]	-.05[f]
Sleep latency	.69[b]	.17[f]
Sleep duration	-.08[f]	.85[a]
Habitual sleep efficiency	.22[f]	.77[a]
Sleep disturbances	.57[c]	.10[f]
Sleep medication use	.45[e]	-.06[f]
Daytime dysfunction	.64[b]	-.11[f]
Percentage of total variance, %	30.2	18.1
Timepoint 3		
Subjective sleep quality	.78[a]	.24[f]
Sleep latency	.76[a]	.14[f]
Sleep duration	.18[f]	.79[a]
Habitual sleep efficiency	.10[f]	.85[a]
Sleep disturbances	.55[c]	.03[f]
Sleep medication use	.40[e]	-.41[e]
Daytime dysfunction	.72[a]	.06[f]
Percentage of total variance, %	34.5	20.0

Factor analysis conducted with maximum likelihood estimation extraction and direct oblimin rotation. a = excellent loadings, b = very good, c = good loading, d = fair loading, e = poor loading, f = loading too low to interpret.
